# Long Non-Coding RNA *Bmdsx-AS1* Effects on Male External Genital Development in Silkworm

**DOI:** 10.3390/insects13020188

**Published:** 2022-02-11

**Authors:** Kai-Xuan Wang, Chun-Bing Chen, Qiu-Xing Wan, Xing-Fu Zha

**Affiliations:** State Key Laboratory of Silkworm Genome Biology, College of Sericulture, Textile and Biomass Sciences, Southwest University, Chongqing 400715, China; wkx7418453@163.com (K.-X.W.); chenchunbingxxc@163.com (C.-B.C.); Wanqiux@outlook.com (Q.-X.W.)

**Keywords:** silkworm, lncRNA, *Bmdsx-AS1*, *BmAbd-B*, EGFR signaling, external genitalia

## Abstract

**Simple Summary:**

LncRNAs are a class of non-coding RNAs longer than 200 nt that are involved in a variety of biological processes. Studies on lncRNAs in *Bombyx mori* have shown that some lncRNAs are involved in brain development, silk production and the response to virus infection of the host. However, the roles of lncRNAs are still largely unknown in the silkworm. In this study, we analyzed the function of lncRNAs *Bmdsx-AS1* in silkworm by transgenic overexpression, which not only affects the development of male silkworm external genitalia, but also participates in the regulation of EGFR signaling pathway. Moreover, we studied the upstream promoter of *Bmdsx-AS1* and found that the *BmAbd-B* transcription factor of the Hox gene family can negatively regulate the expression of *Bmdsx-AS1*. These results laid a substantial foundation for in-depth study of the function of lncRNAs in the silkworm.

**Abstract:**

Long non-coding RNAs (lncRNAs) have been suggested to play important roles in some biological processes. However, the detailed mechanisms are not fully understood. We previously identified an antisense lncRNA, *Bmdsx-AS1*, that is involved in pre-mRNA splicing of the sex-determining gene *Bmdsx* in the silkworm. In this study, we analyzed the changes in the male external genitalia of transgenic overexpressed *Bmdsx-AS1* silkworm lines and analyzed downstream and upstream responses. We found that *Bmdsx-AS1* transgenic silkworms, compared with wild type, showed more claspers in the male external genitalia. Quantitative real-time PCR (qPCR) results indicated that overexpression of *Bmdsx-AS1* decreased the expression of genes in the EGFR signaling pathway. Knockdown of *Bmdsx-AS1* increased the activity of the EGFR pathway. Through promoter prediction, promoter truncation and electrophoretic mobility shift assay (EMSA) analyses, we found that the protein encoded by the Hox gene *BmAbd-B* specifically binds to the promoter of *Bmdsx-AS1*. Moreover, overexpression of *BmAbd-B* in the silkworm BmE cell line indicated that *BmAbd-B* negatively regulates the mRNA expression of *Bmdsx-AS1*. Our study provides insights into the regulatory mechanism of the lncRNA in the silkworm.

## 1. Introduction

LncRNAs are defined as transcripts of more than 200 nucleotides in length that do not encode functional proteins. They are classified as intergenic transcripts, sense or antisense transcripts and enhancer transcripts [1]. Increasing evidence suggests that lncRNAs play diverse roles in biological processes, including epigenetic regulation, dosage compensation effects, cell cycle regulation and cell differentiation regulation [2,3,4,5].

The silkworm (*Bombyx mori*) is a lepidopteran model insect. The silkworm genome has been extensively studied and fully sequenced with a wide range of genetic tools [6,7,8]. Moreover, transgenic overexpression, gene-specific knockdown and knockout are readily available, including CRISPR/Cas9 mutagenesis and the piggyBac system, which allow for disruption of endogenous gene expression and introduction of foreign genes in a time- and tissue-specific manner, respectively [9,10,11,12]. Previous studies on lncRNAs have mainly focused on vertebrates, such as mice and humans. In the silkworm, several studies have been performed on lncRNA. Wang et al. have characterized the lncRNA iab-1, which is mainly encoded by the intergenic region between *BmAbd-A* and *BmAbd-B* in the Homeobox (Hox) cluster in silkworms, and may be involved in some physiological functions [13]. Taguchi et al. have performed fluorescence differential display screening and identified the novel lncRNA *Fben-1*, which is preferentially expressed in the female brain, and have suggested the possible involvement of nuclear noncoding RNA in sexually dimorphic brain functions [14]. Through various transcriptomic and computational approaches, silkworm lncRNAs have been detected on a large scale. Yu et al. have generated 18 RNA-seq datasets and identified 11,810 lncRNAs for 5556 loci by using a lncRNA identification pipeline [15]. Zhou et al. have performed analysis of lncRNAs in the silk glands of domestic and wild silkworms and have found that lncRNAs may affect the phenotypic variation in silk yield between domestic and wild silkworms, through the post-transcriptional regulation of silk protein [16]. Zhang et al. have used genome-wide transcriptome analysis to identify the differentially expressed lncRNAs in silkworm cells with or without BmNPV infection and found that these lncRNAs participate in the host response to BmNPV infection via interactions with their target genes and miRNAs [17]. Zhou et al. have systematically scanned lncRNAs, by using the available silkworm RNA-seq data and public unigenes, and constructed a comprehensive database of silkworm lncRNAs and miRNAs [18]. 

Although many lncRNA datasets have been generated in the silkworm, their biological functions and underlying mechanisms are not still fully understood. In a previous study, we identified the antisense lncRNA *Bmdsx-AS1*, whose genomic sequence is within *Bombyx mori doublesex* (*Bmdsx*) gene [19]. Knockdown or overexpression of *Bmdsx-AS1* resulted in changes in splicing types of the sex-determining gene *Bmdsx* in the silkworm. In this study, we carried out transgenic overexpression of *Bmdsx-AS1* to detect its biological roles and explore its regulatory mechanism.

## 2. Materials and Methods

### 2.1. Silkworm Breeding and BmE Cell Culture

The silkworm (*Dazao*) and the *B. mori* embryonic cell line (BmE cells) were provided from the State Key Laboratory of Silkworm Genome Biology (Southwest University, Chongqing, China). The larvae of silkworms were reared on fresh mulberry leaves at 25 ± 2 °C with 75% relative humidity. The BmE cells were cultured in Grace medium at 27 °C in an incubator. BmE was used for assay of *BmAbd-B* overexpression, and EMSA of the nucleoprotein and DNA probes of *Bmdsx-AS1* promoter.

### 2.2. Transgenic Vector Construction and Strain Acquisition

*Bmdsx-AS1* was cloned from the silkworm testis and inserted into the transgenic vector piggyBac [3×P3-DsRed-SV40] [20] to obtain the final transgenic vector piggyBac [3×P3-DsRed-SV40, A4-Bmdsx-AS1-SV40]. The transgenic vectors were constructed as described in our previous study [19]. We prepared the transgenic vector piggyBac [3×P3-DsRed-SV40, A4-Bmdsx-AS1-SV40] and the helper plasmid pHA3PIG. In order to obtain the transgenic silkworm strain, we mixed the plasmids of the transgenic vector and the helper vector in the proportion of 1:1, and then injected the mixture into non-diapausing *Dazao* embryos by microinjection technology. Silkworms were raised to the third generation and subjected to screening for red eyes with fluorescence microscopy. Total RNA was extracted with TRIzol reagent (Invitrogen, Carlsbad, CA, USA) from the individuals of transgenic silkworms at the first day of the fifth instar larva to detect the expression of *Bmdsx-AS1* with quantitative real-time RT-PCR (qPCR). Some transgenic silkworms and wild type silkworms were fed until moth stage for observation.

### 2.3. Detection of Genome Extraction and Insertion Sites

First, whole transgenic silkworm larvae were frozen in liquid nitrogen for DNA extraction. Second, the transgenic silkworm genome was extracted with a Tissue DNA Kit (Omega Bio-Tek, Norcross, GA, USA), and 12.5 μg of genomic DNA was digested with Hae III at 37 °C overnight. The digestion products were purified with two volumes of anhydrous ethanol and 0.1 volume of NaAc. The genome was purified by self-ligation using T4 DNA ligase. The ligated product was amplified by PCR. The specific primers for amplification are shown in Supplementary Table S1. The PCR products were purified with a Gel Extraction Kit (Omega Bio-Tek, Norcross, GA, USA). The product was ligated with T4 DNA ligase into the pMD19 vector. Then, Escherichia coli transformation and sequencing were used to detect the insertion site. 

### 2.4. Total RNA Extraction and Quantitative Real-Time RT-PCR (qRT-PCR)

Total RNA was extracted with TRIzol^®^ reagent (Invitrogen, Carlsbad, CA, USA) according to the manufacturer’s protocol. The OD value of RNA was measured by spectrophotometer, and the total RNA with the purity (260/280) of 1.9 to 2.0 was used for further assay. Total RNA template was reverse transcribed into cDNA with a PrimeScript RT reagent Kit with gDNA Eraser (TaKaRa, Shiga, Japan). The synthesized cDNA was diluted to 250 ng/μL as the template of qPCR. qPCR experiments were carried out on ABI7500 Real-Time PCR machine (Applied Biosystems, Foster City, CA, USA). Then qPCR was performed to quantify the RNA levels with NovoStart^®^SYBR qPCR SuperMix Plus (Novoprotein, Shanghai, China). All procedures with instruments and kits were performed according to the manufacturer’s instructions and protocols. The mixture (10.0 μL 2×SYBR Green Realtime Master Mix, 0.8 μL qPCR forward primer, 0.8 μL qPCR reverse primer, 6.4 μL ultrapure H_2_O, 2.0 μL Template) was added to the qPCR reaction plate. Conditions (95 °C, 1 min; 95 °C, 20 s; 60 °C, 1 min, 35–45 cycles in the second and third steps) in the reaction refer to the instructions in the reaction kit. According to previous reports [21], the eukaryotic translation initiation factor 4A (BmMDB probe ID: sw22934) was used as an internal control. The primers used for qPCR are listed in Supplementary Table S1. The results of qPCR experiment were repeated more than three times. After the reaction, we export the data from the ABI7500 Real-Time PCR machine. Finally, we used the 2^−ΔΔCt^ method to analyze data. 

### 2.5. RNA Interference (RNAi)

Double-stranded RNA (dsRNA) was synthesized with a TranscriptAid T7 High Yield Transcription Kit (Thermo Fisher Scientific, Waltham, MA, USA). The dsRNA for EGFP was synthesized and used as a negative control. The T7 promoter site was added to the primers in Supplementary Table S1. First, the DNA fragments were amplified by specific primers through PCR. DsRNA was synthesized by using DNA fragments as templates for 5 h at 37 °C. The digestion products were purified with two volumes of anhydrous ethanol and 0.1 volume of NaAc at −20 °C overnight. Then, 50 μg of dsRNA was injected with capillary glass tubes into female and male silkworms from the integument near the third pair of abdominal legs, at the first day of the fifth instar larva. According to the previous studies [22,23], the RNAi knocking down efficiency was assessed at the 48 h post-injection time point. We detected the mRNA level of *Bmdsx-AS1* after 48 h of Bmdsx-AS1 dsRNA injection. After 48 h injection, total RNA was extracted from the gonads and reverse transcribed into cDNA. The cDNA was used for qPCR analysis. Total RNA was extracted from the mixture of at least three silkworms. DsRNA was treated to 30 silkworms for each experiment (10 silkworms per biological replicate). All silkworms were used for evaluating phenotypes.

### 2.6. Bioinformatics Analyses of Gene Promoters and Prediction of Transcription Factors

To characterize the *Bmdsx-AS1* promoter sequence, we identified the sequence upstream of *Bmdsx-AS1* through NCBI Blast. The entire genome of *Bombyx mori* used was from Xia et al. [6]. The promoter (1469 bp in length) was identified from genomic DNA. To predict transcription factors, we identified *Bomyx mori Abd-B* through an online site (http://jaspar.genereg.net) (22 September 2018). We predicted three sites (−1179 to −1173, −737 to −731, −578 to −572) with highest scores for further study. We truncated the promoter sequence according to the analysis of the three sites.

### 2.7. Construction of a Dual Luciferase Vector

Promoter sequence (−1306 to +161) of *Bmdsx-AS1* was obtained by PCR amplification with *Bombyx mori* genomic DNA. Three truncated fragments (−753 to +161, −643 to +161, −556 to +161) were subcloned from the promoter of *Bmdsx-AS1*. Full-length promoter and three truncated fragments were inserted into T5 Zero vector using pEASY-T5 Zero Cloning Kit (TransGen, Beijing, China). The recombinant T5-zero vector was digested by the enzyme of Kpn I and Bgl II to obtain the target fragment with Kpn I and Bgl II restriction sites. Different target fragments were introduced into the Kpn I and Bgl II restriction sites of the luciferase reporter vector PGL3(IE1-SV40). The internal reference vector was PrL(Vg-SV40). The plasmids were extracted for further experiments. 

### 2.8. Luciferase Reporter Assay

BmE cells, at a density of up to 90%, were seeded onto 24-well cell culture plates. After 24 h, PGL3 and PrL plasmids, and overexpression plasmids p1180-BmAbd-B or p1180-EGFP were concurrently transfected into the BmE cells. Luciferase activity was measured 48 h after transfection with a Dual Luciferase Reporter Assay Kit (Promega, Madison, WI, USA). Fluc and Rluc were measured with a microplate reader (Promega, Madison, WI, USA). The ratio of fluorescent signal of Fluc and Rluc was calculated, which was defined as the relative luciferase activity.

### 2.9. Electrophoretic Mobility Shift Assay (EMSA)

The full-length coding sequence of *BmAbd-B* was amplified with Myc-tagged primers. The target fragment of PCR product was cloned into the basic p1180 expression vector (conserved in our laboratory) to generate the recombinant expression vector p1180-BmAdb-B. The p1180-BmAbd-B and p1180-EGFP plasmids were transfected into BmE cells, respectively. The p1180-EGFP plasmid was a negative control group. Cells were collected 72 h after transfection, and nucleoproteins were extracted with a Nuclear and Cytoplasmic Protein Extraction Kit (Beyotime, Beijing, China). The expression of *BmAbd-B* protein was detected by qPCR analysis. EMSA was conducted to verify the binding between nucleic acid and protein according to the protocol of a chemiluminescence EMSA Kit (Beyotime, Beijing, China). The 5′ termini of the DNA probes were synthesized and labeled with biotin. The mixture of DNA probes and nucleoprotein extract in binding buffer was incubated for 20 min at room temperature. The mixture was loaded on a 5% native acrylamide gel [(29:1) acrylamide-bisacrylamide, 100% glycerol, 10% APS, 10 × TBE, TEMED] in 0.5 × TBE buffer. The 5% native acrylamide gel was run at 100 V on ice. After approximately 2 h of electrophoresis, the gel was transferred onto a nylon membrane and cross-linked by UV. The results were visualized in Bioimage analyzer.

## 3. Results and Discussion

### 3.1. Construction of the Bmdsx-AS1 Overexpression Transgenic Line

**Figure 1:**
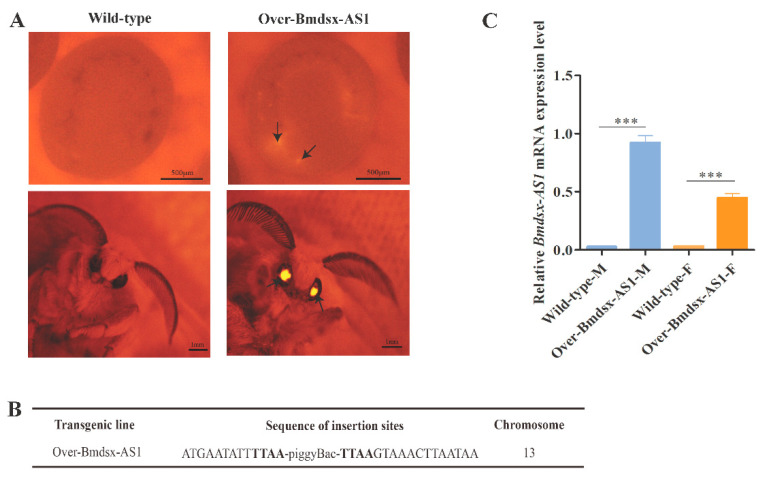
Establishment of the transgenic line Over-Bmdsx-AS1. The transgenic expression vector piggyBac(3×p3-DsRed-SV40, A4-Bmdsx-AS1-SV40) was constructed to overexpress *Bmdsx-AS1*. A4 and 3×p3 were the promoters, and SV40 was used to stop transcription. DsRed is a red fluorescent protein. (**A**) Fluorescent images of G1 egg and moth of the transgenic overexpression of *Bmdsx-AS1* and the wild type. Arrowheads denote the positions of DsRed fluorescence. DsRed was expressed in the compound eyes in transgenic lines, but not the wild type. In order to detect DsRed protein in transgenic lines, photos of silkworm eggs and moths were taken under the red fluorescence of the fluorescence microscope. (**B**) Detection of the insertion site in the transgenic lines. (**C**) The expression level of *Bmdsx-AS1* in transgenic lines and wild types was detected by qPCR. Wild-type-M and Wild-type-F denote the male and female of wild type silkworm, respectively. Over-Bmdsx-AS1-M and Over-Bmdsx-AS1-F denote the male and female of Over-Bmdsx-AS1 transgenic strains, respectively. The expression of *Bmdsx-AS1* was significantly up-regulated in transgenic silkworms. Different numbers of asterisks above the horizontal lines indicate significant differences (*** *p* < 0.001).

In order to detect the biological effects of *Bmdsx-AS1*, the piggyBac-Bmdsx-AS1 vector was mixed with the helper vector pHA3PIG and injected into 400 silkworm eggs within 2 h after egg laying by microinjection. A total of 122 eggs hatched successfully and developed to adults. Through breeding and fluorescent screening of many generations, we obtained transgenic lines with high expression of *Bmdsx-AS1* denoted as Over-Bmdsx-AS1 (Figure 1A). DsRed was expressed in compound eyes of transgenic silkworm, but not in wild-type silkworm. To detect the insertion site in the transgenic lines, we extracted the DNA genomes of transgenic lines and performed inverse PCR. The PCR products were cloned and sequenced. Sequence analysis showed that transgenic *Bmdsx-AS1* was inserted into chromosome 13, and the insertion site was located in the intergenic region (Figure 1B). The closest genes on the left and right of the insertion site were BMSK0007846 and BMSK0007847, with the distance away of 67 kb and 91 kb, respectively. Homologous search results showed both BMSK0007846 and BMSK0007847 are uncharacterized protein genes. To investigate the expression level of *Bmdsx-AS1* in the Over-Bmdsx-AS1 transgenic line, we extracted total RNA from transgenic and wild-type silkworms on day 3 of the fifth instar larva, and reverse transcribed it into cDNA. We used cDNA as the qPCR template to detect the expression level of *Bmdsx-AS1* in the transgenic line. The results showed that *Bmdsx-AS1* expression was significantly increased in both sexes of the transgenic line than in the wild type (Figure 1C). Thus, we confirmed that the transgenic silkworm line Over-Bmdsx-AS1 over-expressed *Bmdsx-AS1*.

### 3.2. Phenotype of Over-Bmdsx-AS1 in Genitals

**Figure 2:**
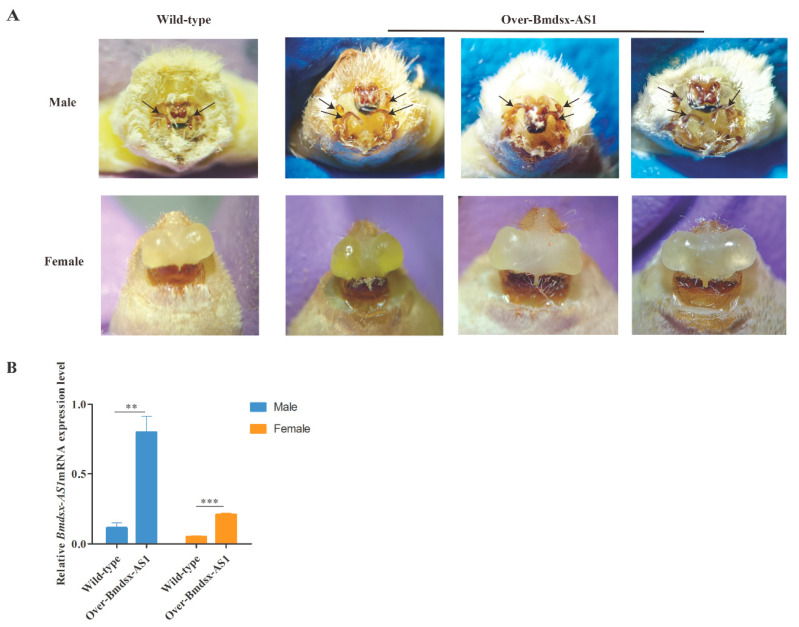
Phenotypic observation of Over-Bmdsx-AS1 strains and expression of transgenic *Bmdsx-AS1* in the external genitalia. (**A**) Changes in external genital morphology in the transgenic line compared with the wild type. In the wild-type silkworm, there are two claspers in the external genitalia. Interestingly, the number of external genital claspers was greater in male transgenic moths. Black arrows represent the clasper. (**B**) Expression of *Bmdsx-AS1* was higher in the external genitalia in the transgenic line. There is a tremendous difference in the expression level of *Bmdsx-AS1* in the external genitalia between Over-Bmdsx-AS1 males and the Over-Bmdsx-AS1 females. The mRNA stability and half-life of transgenic *Bmdsx-AS1* were maybe obviously different in the external genitalia between sexes. Different numbers of asterisks above the horizontal lines indicate significant differences (** *p* < 0.01, *** *p* < 0.001).

To further detect the phenotype of Over-Bmdsx-AS1 strain, we fed the transgenic lines until the moth stage. The morphology was observed, and 15 of the 27 male transgenic silkworms had abnormal external genitals. In transgenic males, three or even four claspers were observed in the external genitalia (Figure 2A). However, there are normally two claspers in wild-type males. Compared with the wild type, the male transgenic lines showed a developmental disorder, displaying more claspers, in the external genitalia (Figure 2A). There is no obvious difference in female external genitalia in the transgenic lines and the wild type. Moreover, we extracted the total RNA from external genitalia and performed qPCR analysis, which indicated that the expression level of *Bmdsx-AS1* was higher in the transgenic lines than the wild type (Figure 2B). We found that the reproductive abilities of the Bmdsx-AS1 overexpression line showed no significant difference than that of wild-type silkworms.

**Figure 3:**
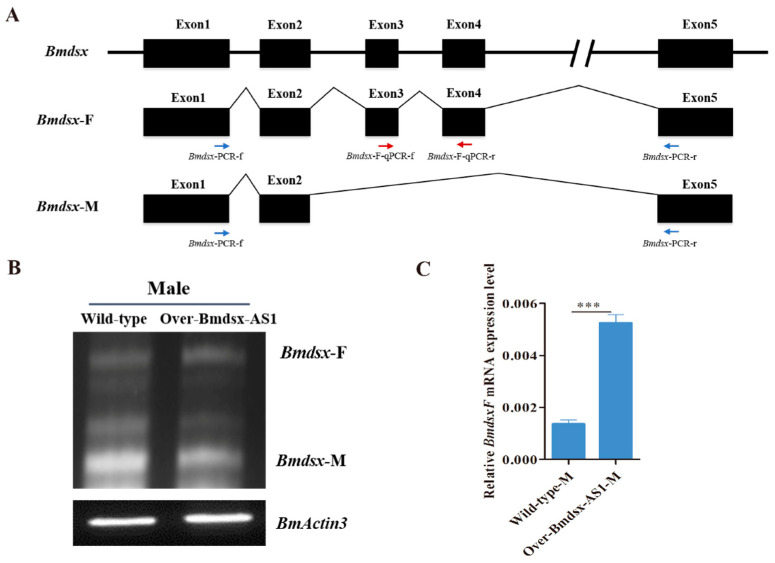
Detection of *Bmdsx* expression in Over-Bmdsx-AS1 transgenic males. (**A**) The gene structure diagram of *Bmdsx* and alternative splicing. Bmdsx-F and Bmdsx-M represent female- and male-type splicing isoform of *Bmdsx*, respectively. The blue and red arrows represent the positions of RT-PCR and qPCR primers, respectively. The transcript of *Bmdsx-AS1* is mainly located in the intron region of the *Bmdsx* gene, and the third exon of *Bmdsx-AS1* overlaps partially the fourth exon of *Bmdsx* with only 7-nt site (GAAAAUG). The primers was designed to avoid the site. (**B**) Expression pattern of *Bmdsx* in the transgenic males by RT-PCR. (**C**) The mRNA level of Bmdsx-F in the transgenic males by qPCR. Different numbers of asterisks above the horizontal lines indicate significant differences (*** *p* < 0.001).

In the silkworm, *Bmdsx* functions as a double-switch gene in the sex-determining cascade [24].The pre-mRNA of *Bmdsx* is alternatively spliced into female- and male-type isoforms (Bmdsx-F and Bmdsx-M, respectively), which encode sex-specific proteins [25]. The sex-specific BmDSX protein promotes male and female sexual differentiation and development [26]. In order to detect the expression pattern of the splicing isoforms of *Bmdsx* in the transgenic males, we carried out RT-PCR experiments by using *Bmdsx* specific primers, Bmdsx-PCR-f and Bmdsx-PCR-r (Figure 3A). The results showed that the expression pattern of Bmdsx-F and Bmdsx-M had no obvious change in the transgenic males compared with the wild-type males (Figure 3B). Exons 3 and 4 of *Bmdsx* specifically exist in female-type splicing isoform, Bmdsx-F, but not in the male-type Bmdsx-M, and the sequence of Bmdsx-M completely overlaps Bmdsx-F (Figure 3A). We could not exclusively detect the expression of Bmdsx-M by qPCR. Thus, we carried out qPCR to detect Bmdsx-F expression by using Bmdsx-F specific primers, Bmdsx-F-qPCR-f and Bmdsx-F-qPCR-r. The results showed that the expression of Bmdsx-F was significantly increased in the transgenic males than that in the wild-type males (Figure 3C). Bmdsx-F includes all the exons of Bmdsx-M. It is interesting that the *Bmdsx-AS1* lncRNA affected expression of male splice form and not Bmdsx-F. This is a probable reason that the lncRNA maybe regulates the splicing process of *Bmdsx* male-specific form, thus affecting expression level of Bmdsx-M.

### 3.3. Overexpression of Bmdsx-AS1 Decreases EGFR Signaling Activity

**Figure 4:**
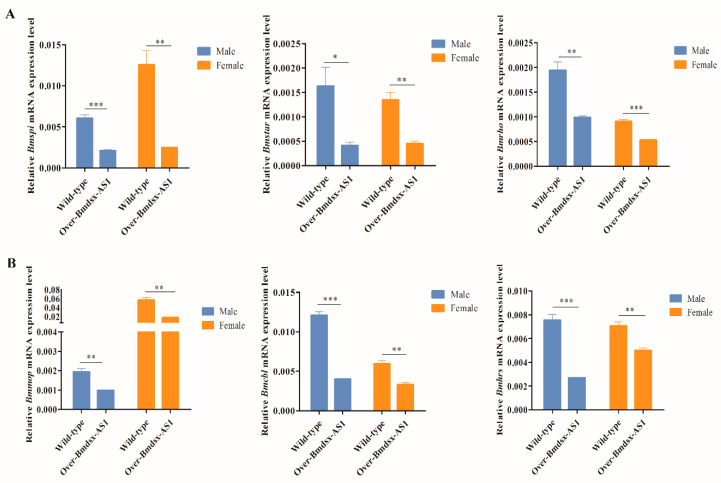
Expression levels of key factors in the EGFR signaling pathway in Over-Bmdsx-AS1 transgenic lines. (**A**) *Bmstar*, *Bmhro* and *Bmspi* were significantly down-regulated in the external genitals in Over-Bmdsx-AS1 transgenic lines. (**B**) Expression of genes associated with endocytosis in the EGFR signaling pathway. The expression of *Bmcbl*, *Bmmop* and *Bmhrs* was significantly down-regulated in Over-Bmdsx-AS1 transgenic lines. Different numbers of asterisks above the horizontal lines indicate significant differences (* *p* < 0.05, ** *p* < 0.01, *** *p* < 0.001).

Development in the moth tail is regulated by the EGFR signaling pathway [27]. To detect expression of genes in the EGFR pathway, we extracted total RNA from the external genitalia of the transgenic Over-Bmdsx-AS1 lines and performed qPCR analysis. *Spi* is an inactive membrane precursor whose processing can induce EGFR activation [28]. *Star* and Rhomboid (*Rho*) proteins are involved in the regulation of *spi* [29]. *Star* is a cargo receptor that transports SPI precursors, and Rho is a protease that cleaves SPI precursors to produce an active and secretory ligand sSPI [30,31]. Additionally, sSPI binds the EGF receptor and induces EGFR dimerization and autophosphorylation, thus leading to EGFR activation [27]. Compared with that in wild type silkworms, the expression of *B. mori star* (*Bmstar*), *B. mori hro* (*Bmhro*) and *B. mori spi* (*Bmspi*) was lower in the external genitalia in the transgenic lines (Figure 4A). The activated EGF receptor exposes the receptor to downstream molecules through endocytosis, thus regulating the EGFR signaling pathway [32,33,34,35]. We analyzed casitas B-lineage lymphoma (*cbl*), myopic (*mop*) and hepatocyte growth factor-regulated tyrosine kinase substrate (*hrs*) genes, which are associated with endocytosis of the EGFR signaling pathway [27]. The expression levels of *B. mori cbl* (*Bmcbl*), *B. mori mop* (*Bmmop*) and *B. mori hrs* (*Bmhrs*) were all lower in the external genitalia in the transgenic lines than in the wild type (Figure 4B). These results indicated that overexpression of *Bmdsx-AS1* decreased the activity of the EGFR signaling pathway.

### 3.4. Knockdown of Bmdsx-AS1 Increases EGFR Signaling Activity

**Figure 5:**
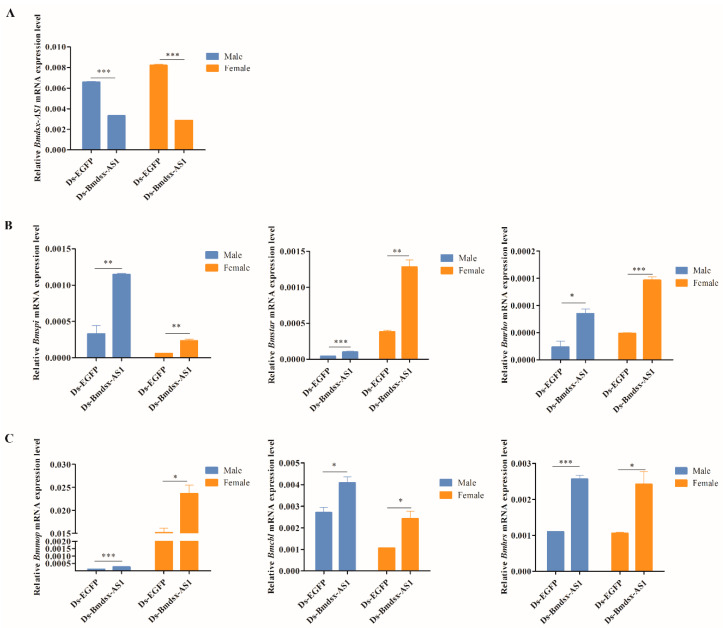
Expression levels of genes associated with EGFR signaling after knockdown of *Bmdsx-AS1*. (**A**) Detection of *Bmds-AS1* expression after RNAi. Ds-EGFP and Ds-Bmdsx-AS1 represent double-stranded RNAs of *EGFP* and *Bmdsx-AS1*, respectively. (**B**,**C**) Expression of *Bmrho*, *Bmstar*, *Bmspi*, *Bmcbl*, *Bmhrs* and *Bmmop*, was up-regulated after *Bmdsx-AS1* RNAi. Different numbers of asterisks above the horizontal lines indicate significant differences (* *p* < 0.05, ** *p* < 0.01, *** *p* < 0.001).

To further determine whether *Bmdsx-AS1* was associated with the EGFR signaling pathway, we performed RNAi experiments in wild-type silkworms. We designed primers to produce synthetic dsRNAs of *EGFP* and *Bmdsx-AS1*, and dsEGFP and dsBmdsx-AS1 were transfected into silkworms, respectively. Because *Bmdsx-AS1* is mainly located in the intron region of the *Bmdsx* gene and only the third exon of *Bmdsx-AS1* overlaps partially transcripts of *Bmdsx*, the primers for dsRNA were designed to locate in the first exon of *Bmdsx-AS1*, thus avoiding the overlapping region. We then performed qPCR to detect the expression of *Bmdsx-AS1* and genes associated with the EGFR pathway. Compared with the control (dsEGFP group), the expression of *Bmdsx-AS1* was significantly lower in the dsBmdsx-AS1 group, which indicated that *Bmdsx-AS1* was knocked down by RNAi (Figure 5A). Meanwhile, the expression of *Bmrho*, *Bmstar*, *Bmspi*, *Bmcbl*, *Bmhrs* and *Bmmop* was elevated in the dsBmdsx-AS1 group. The results showed that the expression of six genes in the EFGR signaling pathway was up-regulated when *Bmdsx-AS1* was knocked down (Figure 5B,C). RNAi phenotype was not observed in the dsBmdsx-AS1 group. The probable reason is that the RNAi knocking-down efficiency of *Bmdsx-AS1* seems not ideal. The previous studies also showed that the high efficiency of RNAi on some genes in the silkworm is difficult to achieve [36,37].

### 3.5. Promoter of Bmdsx-AS1 Contains BmAbd-B Cis-Element

**Figure 6:**
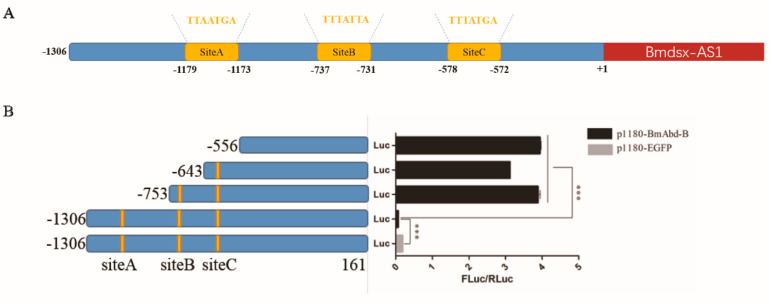
Effect of BmAbd-B on *Bmdsx-AS1* promoter. (**A**) *Bmdsx-AS1* promoter diagram. Yellow boxes represent the predicted potential binding sites of BmAbd-B. The red box represents the sequence of *Bmdsx-AS1*, and the first base of *Bmdsx-AS1* is +1. The promoter of *Bmdsx-AS1* also included the binding sites of other transcription factors, such as *hunchback*, *Broad-complex* and *cf2*. Our study focus on the binding site of the Hox gene *Abd-B*. (**B**) Activities of different truncated *Bmdsx-AS1* promoters. Five experimental groups were designed, pBmdsx-AS1(−1306–+161)-luc and p1180-EGFP, pBmdsx-AS1(−1306–+161)-luc and p1180-BmAbd-B, pBmdsx-AS1(−753–+161)-luc and p1180-BmAbd-B, pBmdsx-AS1(−643–+161)-luc and p1180-BmAbd-B, pBmdsx-AS1(−556–+161)-luc and p1180-BmAbd-B. The vectors of these five groups were transfected into the BmE cells, respectively. The treated cells above were collected 48 h after transfection to measure *Bmdsx-AS1* promoter activity. Blue boxes and yellow boxes represent the truncated promoters and the binding sites, respectively. The ratio of Firefly/Renilla luciferase activities (FLuc/RLuc) represents the promoter activity (*** *p* < 0.001).

To further investigate the regulatory factors upstream of *Bmdsx-AS1*, we carried out promoter analysis of *Bmdsx-AS1*. Interestingly, we found *BmAbd-B* cis-elements in the promoter region of *Bmdsx-AS1*. *BmAbd-B*, a Hox gene, is associated with external genital development [38]. According to analysis of binding sites to BmAbd-B transcription factor, there are three sites with highest scores for further study, SiteA, SiteB and SiteC (Figure 6A). To test promoter analysis, dual luciferase reporter assays were performed. The pBmdsx-AS1(−1306–+161)-luc and p1180-BmAbd-B plasmids were co-transfected into the BmE cells and p1180-EGFP was used as a control. The results showed that *Bmdsx-AS1* promoter activity was down-regulated when *BmAbd-B* was over-expressed (Figure 6B). Furthermore, we confirmed the results by truncating the *Bmdsx-AS1* promoter. The promoter activity increased when we truncated the *BmAbd-B* response element SiteA (−1306 to −753) (Figure 6B). Additionally, the promoter activity similarly increased when the SiteB or/and SiteC were further truncated (−753 to −643, −643 to −553). 

### 3.6. BmAbd-B Protein Specifically Binds to Promoter of Bmdsx-AS1

**Figure 7:**
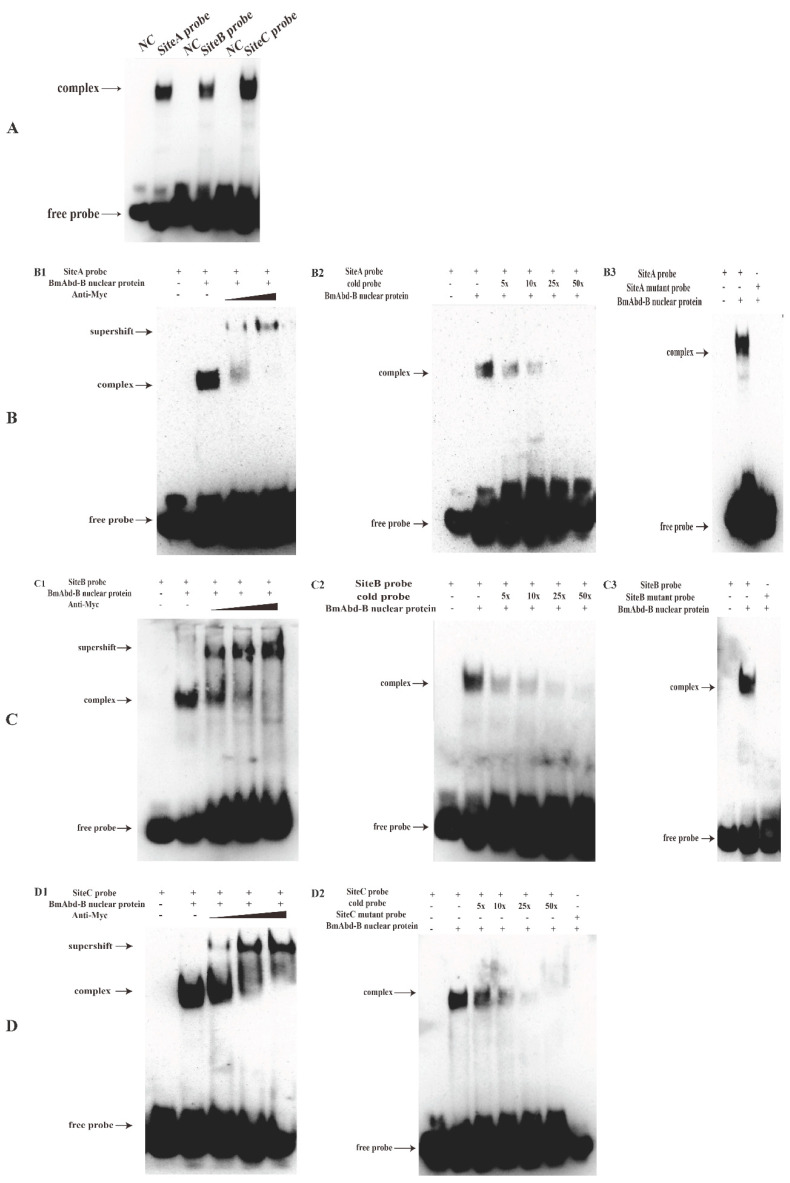
Analysis of the BmAbd-B protein specifically binding to the promoter sequence of *Bmdsx-AS1* by EMSA. (**A**) EMSA of the BmAbd-B protein and the DNA probes (SiteA, SiteB or SiteC) was performed to verify the binding of the protein to the probes. The biotin labeled probes (SiteA, SiteB or SiteC) were incubated without proteins (lane 1, 3, 5) or with nucleoprotein (lane 2, 4, 6), respectively. NC represents the negative control. (**B**–**D**) Gel-shift assay of the BmAbd-B protein and the DNA probe was performed to verify the specific binding of the protein to the probe. (**B1**) Different concentration of anti-Myc antibody (lanes 3 and 4) incubated with the SiteA probe and BmAbd-B nuclear protein extract. The recombinant Myc-tagged BmAbd-B protein was over-expressed in the BmE cells. The positions of the supershift, free and complex probes were shown in arrows. The similar assay was performed for the probes SiteB and SiteC, respectively (**C1**,**D1**). (**B2**) The binding assay of BmAbd-B nuclear protein extract to the probe SiteA with 5-, 10-, 25-, 50-fold excess cold probes (lanes 3–6). The signal of the bound complex became weaker when the cold probe was added. The similar assay was performed for the probes SiteB and SiteC, respectively (**C2**,**D2**). (**B3**) BmAbd-B nuclear protein was incubated with the mutant SiteA probe (lane 3), and the wild-type SiteA probe worked as the positive control (lane 2). The signal of the bound complex did not appear as the probe was mutated. The similar assay was performed for the probes SiteB and SiteC, respectively (**C3**,**D2**). Symbols + and – represent adding the corresponding substance or not.

To detect whether BmAbd-B binds to the promoter of *Bmdsx-AS1*, we performed EMSA to verify BmAbd-B binding to the potential binding sites, SiteA, SiteB and SiteC. According to the sequences of the sites, three Biotinylated DNA probes were designed and synthesized, respectively. Subsequently, we constructed the over-expression vector of *BmAbd-B* (p1180-Myc-BmAbd-B) with Myc-tag to verify the relationship between BmAbd-B and SiteA, SiteB and SiteC, by EMSA. The results showed that the BmAbd-B protein bound to all three sites (Figure 7A). Moreover, we carried out supershift, cold probe competition and mutant probe competition experiments, and found that BmAbd-B bound specifically to the three sites (Figure 7B). Our results indicated that BmAbd-B specifically binds to the promoter of *Bmdsx-AS1*.

### 3.7. BmAbd-B Regulates Expression of Bmdsx-AS1

**Figure 8:**
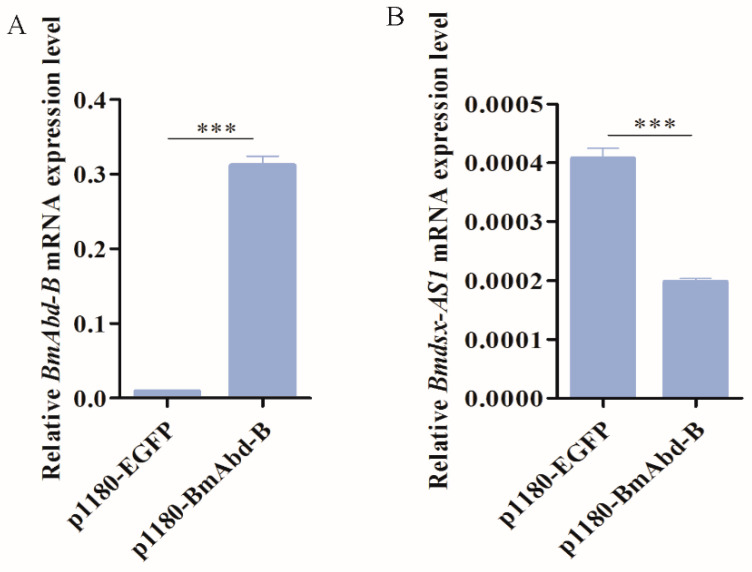
Effect of *BmAbd-B* overexpression on expression level of *Bmdsx-AS1* in cells. (**A**) *BmAbd-B* was over-expressed in the BmE cells. The p1180-EGFP group was used as the control. (**B**) Expression of *Bmdsx-AS1* was down-regulated in the p1180-BmAbd-B group. Different numbers of asterisks above the horizontal lines indicate significant differences (*** *p* < 0.001).

To further verify whether *BmAbd-B* regulates *Bmdsx-AS1*, we performed qPCR analysis of the expression of *Bmdsx-AS1* when *BmAbd-B* was over-expressed in the cell lines. The p1180-BmAbd-B plasmids were transfected into the BmE cells and p1180-EGFP was used as a control. Results showed that the expression of *BmAbd-B* was significantly greater in the p1180-BmAbd-B group than the control group (Figure 8A), which indicated that *BmAbd-B* was successfully over-expressed. Subsequently, the expression of *Bmdsx-AS1* was significantly down-regulated in the p1180-BmAbd-B group (Figure 8B). These results indicated at *BmAbd-B* inhibits the transcription of *Bmdsx-AS1*, thus resulting in decreased expression of *Bmdsx-AS1*.

LncRNAs play critical roles in transcriptional control, physiological processes and epigenetic gene regulation [2,3,4,5]. In a previous study, we found that *Bmdsx-AS1* regulates alternative splicing of *Bmdsx* [19]. In this study, we found that *Bmdsx-AS1* affects male external genital development (Figure 2A). The morphology of external genitalia was altered, and the number of claspers in males was increased in the *Bmdsx-AS1* overexpression transgenic line compared with the wild type. 

EGFR signaling pathway activity has important roles in the extra eighth abdominal segment (A8) in male silkworms [27]. Up-regulation of *spi* expression activates the EGFR signaling pathway [27]. In *Drosophila*, *hro* induces activation of the EGFR signaling pathway [39]. In the EGFR signaling pathway, *Star* is responsible for transporting *spi* precursors [30,40]. In Over-Bmdsx-AS1 transgenic males, the expression of *Bmrho*, *Bmspi* and *Bmstar* was diminished (Figure 4A). The results suggested that, owing to the down-regulation of *Bmstar*, the ability of *Bmstar* to transport *spi* precursor was reduced. In contrast, the expression of *Bmrho* was decreased, and fewer *spi* EGFR ligands were produced, thus decreasing EGFR signaling activity. *Cbl*, *mop* and *hrs* affect the internalization of signaling pathway receptors, endosomal transport and classification of lysosomal degradation [32,33,35]; moreover, down-regulation of *Bmcbl*, *Bmmop* and *Bmhrs* leads to abnormal EGFR signaling in the external gonads. The results of knockdown of *Bmdsx-AS1* were opposite from the overexpression results (Figure 5). In our study, due to the lack of direct experimental evidence for the biological effect of the EGFR signaling pathway on male external genital, further study was needed to confirm the function of the EGFR signaling pathway in the silkworm. Interestingly, expression of Bmdsx^M^ increases the expression of *Bmspi* and activates EGFR signaling in female transgenic silkworms [27]. Activation of the EGFR signaling pathway is beneficial to the development of the A8 abdominal segment in silkworms [27]. Moreover, our previous study has shown that *Bmdsx-AS1* regulates the splicing of *Bmdsx* [19]. We infer that *Bmdsx-AS1* affects the EGFR signaling pathway by regulating expression of *Bmdsx.*

In the previous study, overexpression of *Bmdsx-AS1* in female silkworms showed that male-type splicing isoform of *Bmdsx* arose in females [19]. In the silkworm, the primary determiner of sex is a single female-specific piRNA [41]. Additionally, *Bmdsx* is alternatively spliced into sex-specific mRNAs to encode female-specific (BmDSXF) and male-specific (BmDSXM) proteins [25]. The BmDSX proteins promote silkworm sexual differentiation [26], and there is a competitive antagonism between BmDSXF and BmDSXM in sexual differentiation and development. Although male-type splicing isoform, Bmdsx-M, arose in the transgenic females, the shift of *Bmdsx* sex-specific splicing from female to male model was insufficient and female-type isoform (Bmdsx-F) still remained in the transgenic females [19]. This is a probable reason that no abnormalities were observed in the external genitalia of the Over-Bmdsx-AS1 females in this study.

The Hox gene *BmAbd-B* is a major regulatory factor in the posterior abdomen of the genitalia [27,38]. In our study, overexpression of *BmAbd-B* decreased the transcriptional activity of the *Bmdsx-AS1* promoter (Figure 8). When the binding site SiteA was truncated, the promoter activity of *Bmdsx-AS1* increased significantly (Figure 6B). These results indicated that the SiteA is an important binding site in the promoter region. *BmAbd-B* inhibited the activity of the *Bmdsx-AS1* promoter. Overexpression of *BmAbd-B* led to a decrease in expression of *Bmdsx-AS1* in the BmE cells. In addition, EMSA experiments showed that BmAbd-B specifically binds to the promoter region of *Bmdsx-AS1* (Figure 7). These results suggest that the Hox gene *BmAbd-B* is specific to the promoter of *Bmdsx-AS1* and negatively regulates the transcription of the lncRNA. Precise regulation of Hox genes is essential to achieve proper control of body segments. In many species, *Abd-B* and its homologues have been found to produce sexual dimorphism [42,43,44]. In *Drosophila*, the Hox gene *Abd-B* and the sex-determining gene *dsx* collaborated on regulating the sexually dimorphic abdominal morphology [45]. *Abd-B* is also important for the development of abdominal and genital regions [46,47]. In silkworms, *BmAbd-B* has a positive role in the male A8 and genitalia [27]. Our study showed that *BmAbd-B* regulates the expression of *Bmdsx-AS1*, an antisense lncRNA overlapping with the sex-determining gene *Bmdsx* in silkworms. Our work provides new insight into the interaction between the Hox genes and sex-determining genes. 

## Data Availability

The data presented in this study are available on request from the corresponding author.

## Supplementary Materials

Supplementary Materials can be found at https://www.mdpi.com/article/10.3390/insects13020188/s1. The Supplementary Table S1: List of primer sequences used in this study.

## Abbreviations

dsx	Doublesex
Abd-B	Abdominal-B
SV40	Terminator of *Simian virus 40*
M-MLV	Moloney Murine Leukemia Virus
PCR	Polymerase Chain Reaction
MBP	Maltose binding protein
DsRed	*Discosoma sp.* Red Fluorescent Protein
BmE	Embryonic cells of silkworm
EGFP	Enhanced green fluorescent protein
hrs	Hepatocyte growth factor-regulated tyrosine kinase substrate
rho	Rhodopsin
spi	spitz
cbl	Cbl proto-oncogene
EGFR	Epidermal Growth Factor Receptor
RTK	Receptor Tyrosine Kinase
qPCR	Quantitative Real-Time PCR
EMSA	Electrophoretic mobility shift assay
