# Supplementary material for: Long Non-Coding RNA Bmdsx-AS1 Effects on Male External Genital Development in Silkworm
Source: Insects. 2022 Feb 11;13(2):188. doi: 10.3390/insects13020188 (PMC8875567; doi:10.3390/insects13020188)
Supplement: Supplementary file 1 [file insects-13-00188-s001.zip › insects-1572108-supplementary.pdf]

Table S1 List of primer sequences used in this study.

Primer name	Primer sequence (5′to3′)	Primer purpose
AS1-F	CACAGCGTTGGACGAAAGGAA	qPCR
AS1-R	ACCGGACGAAGGTGGAGAAAT	qPCR
Promoter-AS1-F	GGTACCAGATCTCTGAGGGTTCTGTCGA	Construction of vector
Promoter-AS1-R	AGATCTATACCCATGTTCCTGTTCCGCA	Construction of vector
Bmdsx-AS1-F	CGGGATCCGACACATTTTAGTGTCGGAGATAAAA	Construction of vector
Bmdsx-AS1-R	AAGGAAAAAAGCGGCCGCATTGTTTTGAATGCTGCATCTACAATGAT	Construction of vector
Promoter--754-F	GGTACCAAGCTTAAGGTTACGTTTTATTAA	Construction of vector
Promoter--643-F	GGTACCCTGATTAAAGGATAATCTTCGGT	Construction of vector
Promoter--556-F	GGTACCCGAGTTCACATAACTCTCAGCCAG	Construction of vector
T7-Bmdsx-AS1-F	GTAATACGACTCACTATAGGGGACACATTTTAGTGTCGGAGATAAAAA	RNAi
T7-Bmdsx-AS1-R	GTAATACGACTCACTATAGGGCGACATCGCGAAACAAAGTCTTCAA	RNAi
T7-EGFP-F	AATACGACTCACTATAGGACGTAAACGGCCACAAGTTC	RNAi
T7-EGFR-R	AATACGACTCACTATAGGTGCTCAGGTAGTAGTGGTTGTCG	RNAi
Abd-B-F	CTATCCTCCAGATGCTCCCG	qPCR
Abd-B-R	ACCCTGATGACAGCCTCCAT	qPCR
SPI-F	ACTGTGAGTGTCAAAGCGGGTAT	qPCR
SPI-R	GGACGCAGTCTCCATCATCAG	qPCR
Cbl-F	CGAAAACGACAAGGACATCAG	qPCR
Cbl-R	AATCAATTTGCCACGCAGTG	qPCR
Rho-F	GAGATCGGAAGTATTATCAGGAGC	qPCR
Rho-R	CCAACTCTAACAGTGTAACGCAGA	qPCR
Mop-F	TTGGTGCCCTAAATACCGAACT	qPCR
Mop-R	CTGGGCTTGACCTAAGCAAAT	qPCR
Hrs-F	GACCGGAACTATTGGGAGCA	qPCR

Hrs-R	GTGGAGGCAGTGGAAGCAG	qPCR
